# The SBRT database initiative of the German Society for Radiation Oncology (DEGRO): patterns of care and outcome analysis of stereotactic body radiotherapy (SBRT) for liver oligometastases in 474 patients with 623 metastases

**DOI:** 10.1186/s12885-018-4191-2

**Published:** 2018-03-13

**Authors:** N. Andratschke, H. Alheid, M. Allgäuer, G. Becker, O. Blanck, J. Boda-Heggemann, T. Brunner, M. Duma, S. Gerum, M. Guckenberger, G. Hildebrandt, R. J. Klement, V. Lewitzki, C. Ostheimer, A. Papachristofilou, C. Petersen, T. Schneider, R. Semrau, S. Wachter, D. Habermehl

**Affiliations:** 1 University Hospital Zürich, Department of Radiation Oncology, University of Zurich, Rämistrasse 100, 8091 Zurich, Switzerland; 2 Department of Radiation Oncology, Strahlentherapie Bautzen, Bautzen, Germany; 3 Department of Radiation Oncology, Krankenhaus Barmherzige Brüder, Regensburg, Germany; 4 RadioChirurgicum CyberKnife Südwest, Radiation Oncology, Göppingen, Germany; 5 Department of Radiation Oncology, Universitätsklinikum Schleswig-Holstein, /Lübeck, Kiel, Germany; 6 University Hospital Mannheim, Department of Radiation Oncology, University of Heidelberg, Mannheim, Germany; 7 Department of Radiation Oncology, University Hospital Freiburg, Freiburg, Germany; 8 Department of Radiation Oncology, Klinikum rechts der Isar- Technische Universität München, Munich, Germany; 9 Department of Radiation Oncology, University of Munich – LMU Munich, Munich, Germany; 10 Department of Radiation Oncology, University Hospital Rostock, Rostock, Germany; 11 Department of Radiation Oncology, Leopoldina Hospital Schweinfurt, Schweinfurt, Germany; 12 Department of Radiation Oncology, University Hospital Würzburg, Würzburg, Germany; 13 Department of Radiation Oncology, University Hospital Halle, Halle, Germany; 14 Department of Radiation Oncology, University Hospital Basel, Basel, Switzerland; 15 Department of Radiation Oncology, University Medical Center Hamburg-Eppendorf, Hamburg, Germany; 16 Department of Radiation Oncology, Strahlenzentrum Hamburg, Hamburg, Germany; 17 Department of Radiation Oncology, University Hospital of Cologne, Cologne, Germany; 18 Klinikum Passau, Radiation Oncology, Passau, Germany; 19 Department of Radiation Oncology, University Hospital Heidelberg, Heidelberg, Germany

**Keywords:** Stereotactic body radiotherapy, Liver oligometastases, Outcome, Treated metastases control, Oligometastases, Oligo-recurrence, Sync-oligometastases

## Abstract

**Background:**

The intent of this pooled analysis as part of the German society for radiation oncology (DEGRO) stereotactic body radiotherapy (SBRT) initiative was to analyze the patterns of care of SBRT for liver oligometastases and to derive factors influencing treated metastases control and overall survival in a large patient cohort.

**Methods:**

From 17 German and Swiss centers, data on all patients treated for liver oligometastases with SBRT since its introduction in 1997 has been collected and entered into a centralized database. In addition to patient and tumor characteristics, data on immobilization, image guidance and motion management as well as dose prescription and fractionation has been gathered. Besides dose response and survival statistics, time trends of the aforementioned variables have been investigated.

**Results:**

In total, 474 patients with 623 liver oligometastases (median 1 lesion/patient; range 1–4) have been collected from 1997 until 2015. Predominant histologies were colorectal cancer (*n* = 213 pts.; 300 lesions) and breast cancer (*n* = 57; 81 lesions). All centers employed an SBRT specific setup. Initially, stereotactic coordinates and CT simulation were used for treatment set-up (55%), but eventually were replaced by CBCT guidance (28%) or more recently robotic tracking (17%). High variance in fraction (fx) number (median 1 fx; range 1–13) and dose per fraction (median: 18.5 Gy; range 3–37.5 Gy) was observed, although median BED remained consistently high after an initial learning curve. Median follow-up time was 15 months; median overall survival after SBRT was 24 months. One- and 2-year treated metastases control rate of treated lesions was 77% and 64%; if maximum isocenter biological equivalent dose (BED) was greater than 150 Gy EQD2Gy, it increased to 83% and 70%, respectively. Besides radiation dose colorectal and breast histology and motion management methods were associated with improved treated metastases control.

**Conclusion:**

After an initial learning curve with regards to total cumulative doses, consistently high biologically effective doses have been employed translating into high local tumor control at 1 and 2 years. The true impact of histology and motion management method on treated metastases control deserve deeper analysis. Overall survival is mainly influenced by histology and metastatic tumor burden.

## Background

Stereotactic body radiation therapy (SBRT) is a dedicated form of external beam radiotherapy which is characterized by a steep dose gradient outside the irradiated tumor volume while escalating the dose inside the target volume. Combined with high precision patient setup and image guidance, this is a highly effective local treatment while achieving a very low toxicity profile. Usually, treatment protocols include higher single doses and the fraction number ranges from a radiosurgery (1 fraction) procedure up to 10 fractions.

Established for treating brain metastases, SBRT has been expanded to treat tumors and metastases at numerous body sites including the lung, liver, bone and prostate. Most experience has been gathered so far for the treatment of NSCLC stage I in inoperable patients where it is now considered standard of care [1].

Very early, SBRT has been evaluated in patients with lung and liver oligometastases as well, though published series only report small patient numbers and different treatment protocols. Recently, with the widespread adoption of the concept of oligo-metastases [2], interest has been increased and larger series have been published [3]. In addition, the terms of oligo-recurrence (metastases detected while primary tumor controlled) and sync-oligometastases (primary tumor and limited number of metastases detected simultaneously) have been coined to depict the occurrence of metastases in the course of oligometastasic disease [4, 5].

In contrast to lung tumors where consistent rates of treated metastases control above 85% have been reported in recent studies, results with regard to treated metastases control of liver lesions are more heterogeneous ranging from 60 to 100% and 55%–90% at 1 and 2 years, respectively [6–13]. Currently, SBRT is mainly being used if a patient is not medically fit for surgery, not technically resectable or declines surgical intervention as well as if other local therapies like radiofrequency ablation are not possible due to size or location near larger vessels, and is has been added to the possible armentarium of local therapies to be considered besides resection in metastasized CRC [14, 15]. Still, results of liver SBRT are very encouraging especially considering the larger size and the critical location of the lesions compared to other local ablative therapies [8, 10, 16, 17].

Patient selection for treatment is a critical issue. Most benefit regarding progression free survival and possibly overall survival is expected in the state of oligo-recurrence, as witnessed in small prospective series for oligometastatic NSCLC [18, 19]. In addition, the recently presented outcome data of the EORTC-NCRI CCSG-ALM Intergroup 4004 trial could demonstrate in a randomized fashion a positive effect of a local ablative therapy in the form of radiofrequency ablation on OS in patients with liver oligometastases from CRC in addition to chemotherapy [20]. Currently, up to 4 metastases is considered a safe and reasonable number to treat [7], although most series reported a median number of metastases treated of one. Patients should be in a reasonable performance status (KPS ≥70) and the projected survival should be beyond 6 months. The optimal treatment, including the use of SBRT, should be ideally discussed and recommended in a multidisciplinary tumor board [14].

Still, relevant questions remain open regarding optimal patient selection, radiation dose and fractionation or radio-sensibility of different histologies which cannot be answered satisfactorily with the current available data due to the small sample size of the prospective and retrospective reports.

The intent of this pooled analysis is to set-up an outcome-based database and analyse the pattern of care of liver SBRT in Germany and Switzerland. We herein report on the evolution of SBRT, treatment characteristics as well as outcome with respect to treated metastases control and overall survival in 474 patients with 623 liver oligometastases.

## Methods

### Patient eligibility

All patients treated with SBRT for liver oligometastases after its introduction into the clinic in Germany and Switzerland between 1997 and June 2015 were eligible to be included in this pattern of care analysis. Patient, tumor and treatment characteristics were retrospectively collected and entered in a centralized database. Centers were eligible to provide patient data, if they were performing liver SBRT. No formal inclusion criteria for participation were mandatory.

Inclusion criteria were patients with liver oligometastases from any histology-proven primary solid tumor. Clinical diagnosis was based on radiological imaging without mandatory biopsy of the liver metastasis. Patients receiving liver SBRT were included when they were medically inoperable, presented with non-resectable metastases which were not qualifying for alternative focal treatment such as radiofrequency ablation (RFA) or if they refused invasive therapies. SBRT definition was based on the target volume concept, application of conformal treatment planning and stereotactic or image-guided patient setup as well as hypo-fractionated treatment application. The participating centers used, based on available technology and tumor size and location in correlation to organs-at-risk, a center-specific fractionation schedule.

Patient data was reported anonymously and pooled in a common database by the coordinating center (Department of Radiation oncology, University Hospital of Zurich, Zurich, Switzerland). The database consisted of more than 50 parameters including patient characteristics, primary tumor characteristics and oncological course of disease and further therapies. Additionally, technical data on radiotherapy delivery as well as clinical outcome were collected and further analyzed.

The multicenter data collection, database and analysis was approved by the Ethics committee of the Kanton Zurich, Switzerland (BASEC-Nr. 2016–00744). The data collection of the individual participating centers was approved according to local regulations and approved by the respective local ethics committees.

### Endpoints and toxicity definitions

Local failure of a metastatic lesion was defined as either reappearance after complete remission or re-growth after initial partial response to SBRT in follow-up CT or MRI scans. PET-CT scans were used by some centers in equivocal cases to confirm local recurrence. Extrahepatic tumor status was classified as either no evidence of disease (NED), stable (SD) or progressive disease (PD).

Acute toxicity was scored according to the National Cancer Institute CTCAE v3.0 criteria during and up to 3 months after SBRT. Late toxicity was graded using the RTOG/EORTC criteria.

### Statistical analysis

Actuarial survival time and freedom from local failure (called “treated metastases control”) were calculated according to the Kaplan-Meier method. For univariate and multivariable analysis of prognostic factors (listed in Table 3) the Cox proportional hazard model was used. Comparison of survival between groups was performed using the log-rank test.

For overall survival, any death and for disease specific survival death from the underlying cancer was defined as an event. For actuarial local tumor control, progression of the treated lesion was defined as stated in the methods section. For this endpoint, patients who died from other diseases without tumor regrowth or progression at that time were censored. All time intervals were calculated from the last day of SBRT.

Biological effective doses were calculated according to the LQ formalism:

$$ BED={n}^{\ast }{d}^{\ast}\left(\frac{d}{\raisebox{1ex}{$\alpha $}\!\left/ \!\raisebox{-1ex}{$\beta $}\right.}\right) $$

with n being the number of fractions, d the daily single fraction dose and using an alpha-beta for tumor tissue of 10 Gy.

BED_isocenter_ of 150 Gy_10_ has been determined as descriminator for treated metastases control in a separate ROC analyis (data not shown) and is in line with a previous report from our group [21].

PTV volumes have been used as surrogates for the GTV volumes in the multivariable Cox regression model. This was based on the approach that the results of the multivariable Cox proportional hazard model after imputation of missing GTV values using the R package “mice” were comparable with the respective results after inclusion of PTV instead of GTV (data not shown). As the same variables had been chosen by the model on the respective endpoints, PTV was chosen for use in multivariable cox regression analysis instead of GTV to work with real data, instead of imputed one. Statistical analysis was performed with the R statistical environment version 3.3.1.

## Results

### Patient and tumor characteristics

In total, 17 German and Swiss academic and non-academic centers participated and collected data on 474 patients with a total of 623 metastases. Patient data was collected from university (*n* = 13), public (*n* = 2) and private centers (*n* = 2) in Germany and Switzerland including overall data from 474 patients with 623 liver oligometastases treated with SBRT. The most frequent primary tumor was colorectal cancer (48.1%), followed by breast cancer (13.3%), non-small cell lung cancer (6.1%) and pancreatic cancer (5.1%). In most patients, one liver metastasis was treated (*n* = 369). In the remainder cohort two to four metastases (n = 2: 75 pts.; n = 3, 15 pts.; *n* = 4: 9 pts) have been treated simultaneously. Repeat stereotactic radiotherapy for new liver oligometastases was performed in only 4 patients. Median follow-up was 15 months (range: 1–178 months). SBRT treatment of the liver metastasis was performed at a median time of 27 months (range: 0–392 months) after diagnosis of the primary tumor.

The median number of patients per institution was 13 (range: 2–178), the median number per institution per year was 5 (range: 1–13). Patient and treatment characteristics are summarized in detail in Tables 1 and 2.

**Table 1:** Patient Characteristics

	No. of Patients/ Metastases	Perent	Median	Minimum	Maximum
	474 pts.				
Age (yr)			64	15	93
Sex					
male	268 pts.	56.7%			
female	206 pts.	43.3%			
Pretreatment performance scale (Karnofsky index) (%)			90	40	100
Histology					
Colorectal cancer	228 pts.	48.1%			
Breast cancer	63 pts.	13.3%			
NSCLC	29 pts.	6.1%			
Pancreatic cancer	24 pts.	5.1%			
Others	130 pts.	27.4%			
Chemotherapy prior to SBRT					
Yes	325 pts.	65.6%			
No	80 pts.	16.9%			
Unknown	68 pts.	17.6%			
Number of liver metastases per patient	474 pts. / 623 mets		1	1	4
n = 1	372 pts.	78.5%			
n = 2–4	102 pts.	21.5%			
Status of extrahepatic disease					
Oligo-recurrence group	119 pts.	25.1%			
Sync-oligometastases group	235 pts.	49.6%			
Unknown	120 pts.	25.3%			
Time interval between primary tumor diagnosis and SBRT treatment (months)			27.0	0.0	391.0
Follow-up (months)			15	1	178

**Table 2:** SBRT Treatment Characteristics

	No. of Lesions	Percent	Median	Minimum	Maximum
	623				
GTV volume (ccm)			27	0.6	699
PTV volume (ccm)			71.3	4.5	1074.0
Single fraction dose (PTV encompassing) in Gy			12	2.1	30.0
Single fraction dose (isocenter) in Gy			18.48	2.95	46.9
Total BED (PTV encompassing) in Gy			69.4	10.4	187.5
Total BED (Isocenter) in Gy			125.9	27.2	292.4
Prescription isodose (%)			80.0	42.0	100.0
Most common prescription isodoses					
95% isodose	70	11.2%			
80% isodose	199	31.9%			
65% isodose	131	21.0%			
60% isodose	38	6.0%			
Number of SBRT fractions			3	1	13
1-fraction SBRT	189	30.3			
3-fraction SBRT	207	33.2			
5-fraction-SBRT	158	25.4			
other fractionation schemes	69	11.1			

*GTV* gross target volume, *PTV* planning target volume, *BED* biological effective dose

### Patterns of care

**Fig. 1:**
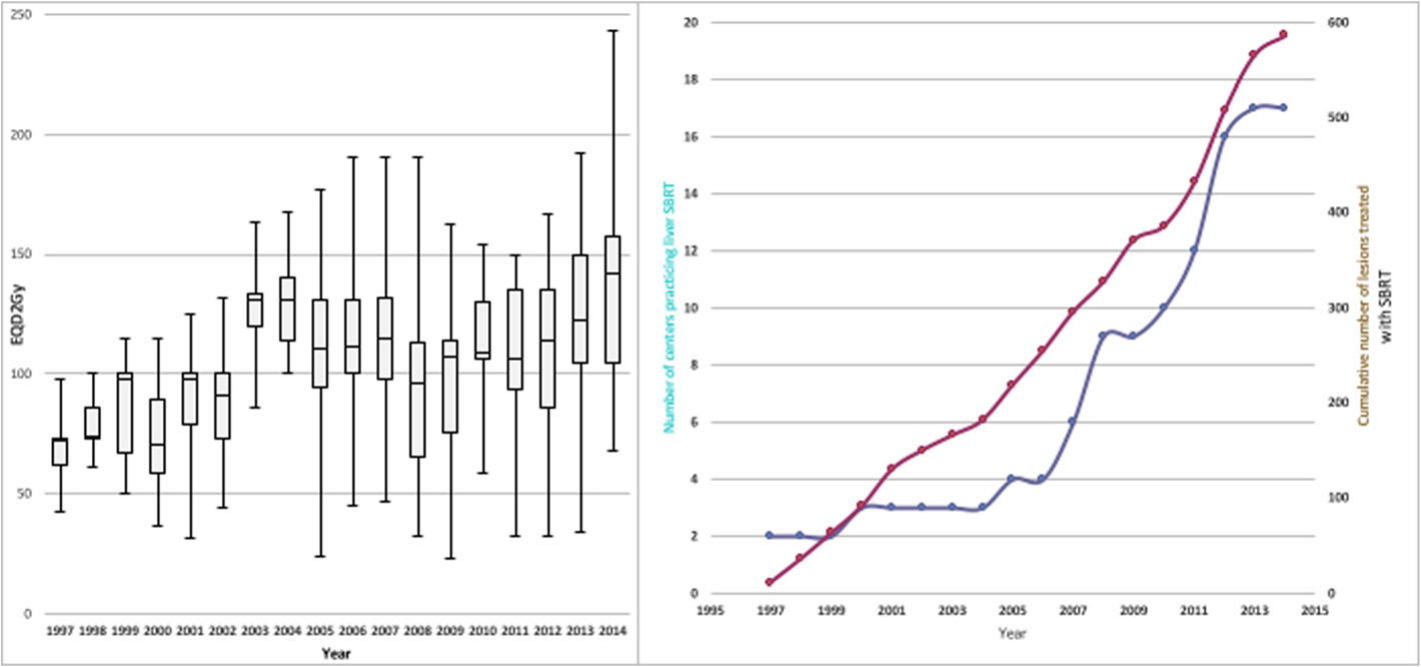
**a** Cumulative number of centers practicing liver SBRT and number of treated lesions from 1997 to 2014. **b** Change of prescribed biologically effective dose (BED) for SBRT liver oligometastases from 1997 to 2014

SBRT for liver oligometastases was first introduced in 1997 and adopted for clinical evaluation by three university centers between 1997 and 2000. All three centers used a vigorous patient immobilization for setup and a stereotactic frame for target localization. General adoption of SBRT for liver oligometastases developed slowly and broader introduction into clinical routine started in 2008 (see Fig. 1a).

#### Time trends in dose prescription

Despite careful introduction into clinical routine, no standardized dose and fractionation protocol has emerged over time. On the contrary, significant interinstitutional variation in the single fraction, total dose prescribed, number of fractions and the respective prescription isodose could be observed (Fig. 1b).

There was as significant time-trend in total dose prescribed in the initial phase after introduction of SBRT. The mean BED_isocenter_ before 2003 was 102.5 Gy, whereas it significantly rose to 134.3 Gy thereafter and remained constant (*p* < 0.001; Fig. 1b).

#### Treated metastases control

Treated metastases control in the total cohort with 607 evaluable metastases was 76.1%,63.8% and 55.7% at 1,2 and 3 years (Fig. 3a). In univariate analysis, maximum BED, histology, systemic therapy before SBRT, GTV and PTV volume were all significant predictors for treated metastases control (Fig. 3b-d; Table 3). Patients receiving systemic therapy before SBRT had a worse treated metastases control rate compared to patient who had received no pretreatment therapy. Metastases from colorectal cancer had a significantly worse treated metastases control rate at one year (67%) compared to breast cancer (91%), NSCLC (88%) or other histologies (80%). Patients treated after 2003 had a significant better treated metastases control rate compared to patients treated before. Local control for metastases treated with advanced motion management methods defined as either gating (active breathing control; free breathing gating) or tracking (fiducial based) was significantly higher compared to methods relying on target localization during free breathing, including CBCT based strategies.

**Table 3:** Univariate and multivariate analysis for local control and overall survival according to patient and tumor characteristics

	Univariate		Multivariate	
	HR (CI)	*p*-value	HR (CI)	*p*-value
Local control				
Prior CTx^a^	2.19 (1.23–3.91)	0.008	2.19 (1.18–4.06)	0.13
Histology (CRC)	1.99 (1.31–3.04)	0.001	1.71 (1.04–2.80)	0.03
Histology (BCa)	0.53 (0.25–1.13)	0.10	0.45 (0.19–1.02)	0.06
Histology (NSCLC)	0.86 (0.26–2.85)	0.81	0.67 (0.19–2.35)	0.54
GTV Volume^c^	1.004 (1.002–1.005)	< 0.001	–	
PTV Volume^c^	1.002 (1.001–1.003)	< 0.001	1.001 (1.00–1.002)	0.003
BED Isocenter^c^	0.993 (0.989–0.997)	< 0.001	0.99 (0.98–1.00)	0.002
BED prescription^c^	0.72 (0.51–1.01)	0.003	1.00 (0.99–1.01)	0.970
Advanced Motion management^a^	0.46 (0.29–0.72)	< 0.001	0.57 (0.33–0.96)	0.04
Before 2003^a^	1.50 (1.04–2.16)	0.031	1.02 (0.61–1.70)	0.932
Overall survival				
KI^c^	0.69 (0.51–0.94)	0.02	0.74 (0.53–0.98)	0.06
Gender	0.80 (0.63–1.01)	0.06	0.76 (0.54–1.06)	0.10
Histology (CRC)	0.61 (0.46–0.79)	< 0.001	0.64 (0.45–0.92)	0.01
Histology (BCa)	0.52 (0.36–0.76)	< 0.001	0.60 (0.36–0.99)	0.05
Histology (NSCLC)	1.42 (0.87–2.33)	0.16	1.14 (0.64–2.07)	0.65
Prior CTx^a^	0.91 (0.65–1.27)	0.58	1.17 (0.76–1.80)	0.47
Extrahepatic status^b^	0.81 (0.60–1.1)	0.18	0.83 (0.49–1.43)	0.52
Solitary^a^	0.73 (0.57–0.92)	0.007	1.12 (0.61–2.03)	0.72
# of liver metastases (1 vs. 2–4)	0.94 (0.72–1.23)	0.65	0.80 (0.52–1.23)	0.32
GTV Volume^c^	1.003 (1.002–1.004)	< 0.001	–	
PTV Volume^c^	1.002 (1.001–1.002)	< 0.001	1.002 (1.001–1.003)	< 0.001
Local recurrence after SBRT^a^	0.88 (0.68–1.51)	0.36	0.82 (0.56–1.19)	0.29
BED isocenter^c^	0.997 (0.994–1.00)	0.06	1.00 (0.99–1.004)	0.97

*CTx* chemotherapy, *BCa* breast cancer, *BED* biologically effective dose, *GTV* gross tumor volume, *PTV* planning target volume, *PS* performance status, *HR* hazard ratio, *CI* 95% confidence interval), *CRC* corolrectal caner, *NSCLC* non-small cell lung cancer

^a^binary coded variables with yes vs. no; ^b^complete remission/stable disease vs progressive disease; ^c^volume and minimum biologically effective dose as continuous variables

As sufficient events were available, all variables investigated in univariate analysis were entered in the multivariable proportional hazard cox regression model. As a considerable proportion of GTV volumes (49%) were missing, PTV volumes have been entered in multivariate analysis instead. Systemic therapy before SBRT, PTV volume, BED_isocenter_, motion management methood, and SBRT treatment before 2003 remained as independently significant variables.

#### Overall survival

**Fig. 2:**
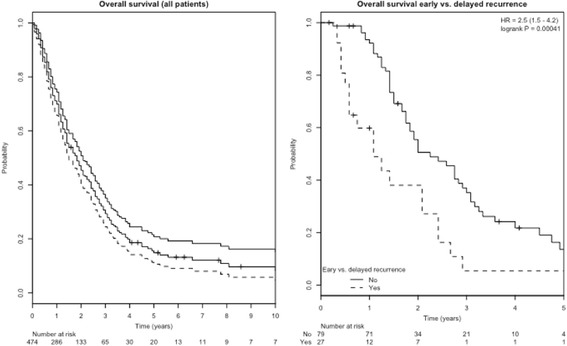
Kaplan-Meier estimated overall survival a) for the whole patient cohort and b) stratified for patients with local recurrence within 6 months

Overall survival in the total cohort was 70%, 29% and 15% at one, 3 and 5 years (Fig. 2a). Seven patients experienced a long-term survival and could be observed at 10 years of follow-up. In univariate Cox regression analysis, KPS, histology (breast and colorectal cancer having a better prognosis) and GTV volume were all significant predictors for overall survival (Table 3). Interestingly, pre-SBRT chemotherapy, number of metastases treated and extrahepatic disease status were not associated with overall survival.

Also, achieving treated metastases control did not influence overall survival in univariate analysis. In contrast, early local recurrence (within 6 months of SBRT) versus late local recurrence was significantly associated with worse survival (Table 3; Fig. 2b).

In multivariable cox regression, only tumor volume, histology (specifically colorectal histology) and early vs. delayed local recurrence remained independent predictors of overall survival.

### Toxicity

Acute toxicity data was available for 73% of the patients (*n* = 347). Grade 1–2 toxicity was observed in 23% and consisted mostly of fatigue, nausea and diarrhea. Grade 3 acute toxicity occurred in less than 1% with one gastric ulcer being the most severe side effect. No toxicity greater grade 3 was observed.

Chronic toxicity data was available for 44% of the patients (*n* = 208) and consisted of fatigue, nausea, diarrhea, liver enzyme elevation and jaundice. Chronic grade 1–2 was observed in 10% and chronic grade 3 toxicity in 1.4% with no chronic toxicity greater than grade 3 reported. Grade 3 toxicity was due to radiation hepatitis with enzyme elevation (*n* = 1), liver fibrosis (*n* = 3; one with consecutive varicosis and bleeding) and necrotic reaction of treated metastases (*n* = 1).

## Discussion

In this mutli-center database centralized data on patient, tumor and treatment characteristics of a large body of patients treated with SBRT for liver oligometastases to describe the evolution and patterns of care of liver SBRT between 1997 and 2015 in Germany and Switzerland has been collected. In addition, outcome data including factors predicting for treated metastases control and overall survival as well as toxicity could be successfully gathered: for treated metastases control, systemic therapy before SBRT, PTV volume, BED_isocenter_, motion management methood, could be identified as independently significant variables; for overall survival, PTV volume, histology (colorectal histology exhibiting a better prognosis) and early vs. delayed local recurrence were independent predictors of overall survival.

Implementation of liver SBRT started in 1997 with CT-simulated frame-based stereotactic approach. With the advent of 4D CT and CBCT based image-guidance in addition to reports on safety and efficacy of liver SBRT, a steady and continued adoption of this technology started in 2005 (Fig. 1a). Over time, more advanced motion management methods were introduced, mainly implemented with robotic tracking systems, and − although scarcely − other motion mitigation approaches like active breathing control or gating.

Although SBRT had been introduced at a high-quality level across all centers, the applied fractionation scheme greatly varied with regards to fractionation and total dose (Table 2). Nevertheless, the applied mean BED_isocenter_ was consistently high and reflects the general notion of the initial dose finding learning curve (Fig. 1b). In these first years, a consistent shift to higher prescription doses was observed around 2003 (Fig. 1b) which translated in a noticeable improvement in treated metastases control. This indicates that centers starting SBRT for liver oligometastases after 2003 did not include a strategy of dose escalation to gain experience, but adopted - if possible in the individual patient situation - an effective BED at the time of individual clinical introduction of SBRT. Inhomogeneous dose prescription - i.e. lower prescription doses at the PTV periphery allowing significantly higher doses to build up towards the iso-center – were applied with the 80%- and 65%- isodoses being the most common prescription isodoses.

Unfortunately, most of the previously published studies only report the PTV prescription dose and no details on the dose distribution within the PTV or GTV, so that a direct comparison of the different doses applied is difficult [8, 10, 11, 22–26]. Therefore, despite a consistent dose response relationship reported in most of these publications, a clear conclusion on the minimally required PTV prescription dose or the relevance of inhomogeneous dose distribution within the PTV to achieve a certain level of treated metastases control is difficult.

**Fig. 3:**
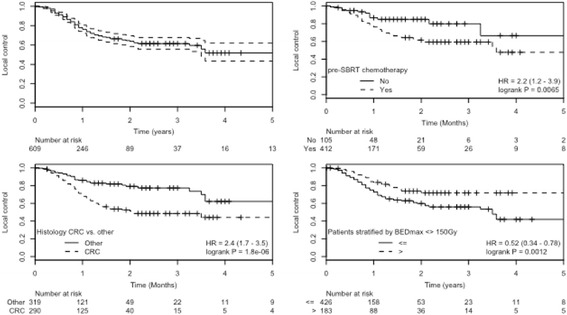
Kaplan-Meier estimated treated metastases control for all patients (**a**) and stratified by (**b**) pre-SBRT chemotherapy, (**c**) histology, (**d**) BED_isocenter_

A clear dose response relationship could be established within our dataset over all histologies for the PTV prescription dose as well as the dose at the isocenter recalculated to the BED using the LQ-formalism. Finally, BED_isocenter_ remained the strongest factor influencing treated metastases control in MVA. Consistent with our dose-response analysis in primary and secondary lung tumors [21], we believe that the dose distribution and the BED within the GTV is clinically more relevant than the minimally PTV prescribed dose and BED_isocenter_ serves as the most robust GTV dose surrogate in this respect. In our liver SBRT cohort, If a BED_isocenter_ of greater than 150 Gy_10_ was applied (Fig. 3d), a treated metastases control of > 80% at 2 years could be achieved which is comparable to the published literature [21].

As reported by other groups, tumor volume (GTV) and planning target volume (PTV) were predictive of local failure as well [27, 28], though this has not been found in other reports [10, 11]. Most interestingly, pre-SBRT chemotherapy and colorectal histology appeared as negative predictive factors for treated metastases control. This has been separately addressed by our group where we could show that the negative influence of pre-SBRT chemotherapy on treated metastases control was the major confounding factor of the inferior treated metastases control in colorectal histology [16]. In contrast, breast cancer histology appears to be more responsive to SBRT irrespective of the pre-SBRT chemotherapy status. Due to the small numbers of patients with breast cancer in our cohort, this finding was only borderline significant in multivariable analysis (Table 3).

Considering the general negative selection of patients which were not considered as surgical candidates, overall survival appears to be reasonable and indicates that longer term survival beyond 5 years is possible after SBRT.

In multivariable only histology, tumor volume and early vs. delayed local recurrence remained independent predictors of overall survival. Interestingly, pre-treatment chemotherapy did not influence overall survival in our cohort. This is in contrast to the recent update of the EORTC 40004 trial where the addition of RFA to systemic therapy improved overall survival and the report by Fode et al. on SBRT for oligo-metastatic solid cancer where pre-SBRT chemotherapy was associated with improved survival as well. Most probably, patient selection is the dominating factor for these contrary findings and reflects the difficulties in comparing different series for oligo-metastatic solid tumors: in our series, 75% of patients had a singular liver oligometastases in contrast to 15% [20] and 50% [29].

The finding is the most intriguing finding: patients developing local recurrence within 6 months compared to later time points had a significant worse overall survival. As developing a local recurrence per se (not taking a time factor into account) had no impact on overall survival, this finding is most probably a reflection of a more aggressive tumor phenotype in these patients and can not necessarily be attributed to the effect of treated metastases control (in the liver) on overall survival. Therefore, it would be desirable to be able to predict the projected individual OS to define which patient would benefit most from local therapies such as SBRT. Further analysis of the dataset will focus on developing predictive models to estimate individual patient’s survival.

Reported toxicity profile for acute and late toxicity was very favorable. Documentation for acute toxicity was very good with 73% and dropped considerable for late toxicity with 44%. We are aware that due to the retrospective nature of the reporting, especially late toxicity scoring must be viewed with caution. Still, no reported grade 4 or 5 toxicity within the follow-up period and a grade 3 rate of less than 2 % seems to be very encouraging and reflects the careful introduction of SBRT with reasonable fractionation schemes for abdominal SBRT.

## Conclusions

After an initial learning curve with regards to total cumulative doses, consistently high biologically effective doses have been employed translating into high local tumor control at 1 and 2 years. Through the continuous technical development, the implementation of advanced motion management techniques such as gating with active breathing control, tracking and use of fiducial markers have increased during the last years and contributed to the improvement in treated metastases control over time while minimizing the excellent therapy-related toxicity profile. Besides radiation dose, tumor volume, pre-SBRT chemotherapy and histology have been identified as predictive factors for treated metastases control. OS is mainly governed by histology and tumor volume. Most intriguingly, local recurrence per se did not influence prognosis, but the time-dependent occurrence: patients with early recurrence within 6 months had a significantly worse OS.

## Abbreviations

BED: Biologically effective dose
CT: Computed tomography
CTCAE: Common Terminology Criteria for Adverse Events
DEGRO: Deutsche Gesellschaft für Radiologische Onkologie
EORTC: Eurpean Organization for Research and Treatment of Cancer
EQD2Gy: Equivalent dose in 2 Gy
Fx: Fraction
GTV: Gross tumor volume
Gy: Gray
NED: No evidence of disease
PD: Progressive disease
PTV: Planning target volume
RFA: Radiofrequency ablation
RTOG: Radiation Therapy Oncology Group
SBRT: Stereotactic body radiation therapy
SD: Stable disease
